# Pembrolizumab-Induced Mobitz Type 2 Second-Degree Atrioventricular Block

**DOI:** 10.1155/2020/8428210

**Published:** 2020-01-28

**Authors:** Alisha Khan, Sana Riaz, Robert Carhart

**Affiliations:** ^1^Department of Medicine, SUNY Upstate Medical University, USA; ^2^Department of Cardiology, SUNY Upstate Medical University, USA

## Abstract

Pembrolizumab is a monoclonal antibody directed towards programmed cell death protein 1 (PD-1) and is an antineoplastic drug which has a growing variety of oncologic uses. Pembrolizumab is commonly associated with immune-related adverse events (IRAEs) but is infrequently noted to cause cardiotoxicities such as myocarditis, arrhythmias, and heart failure. The following case report illustrates the clinical course of a 67-year-old female patient with stage IV non-small-cell lung cancer who developed Mobitz type 2 second-degree atrioventricular block three weeks after receiving her first infusion of pembrolizumab. Within a few hours of presentation, she progressed to symptomatic complete heart block requiring emergent placement of a temporary transvenous pacemaker. The article further discusses proposed mechanisms to explain IRAEs and management of IRAEs. We conclude by recommending a higher degree of caution and awareness among all physicians when treating patients on immunotherapy and a multidisciplinary approach when considering resumption of immune checkpoint inhibitor therapy.

## 1. Introduction

Since its initial approval by the Food and Drug Administration in the United States in 2014, the indications for the use of the antineoplastic drug pembrolizumab have steadily continued to increase in the world of oncology. The drug is an immune checkpoint inhibitor most commonly used in the treatment of melanoma and non-small-cell lung cancer (NSCLC).

Pembrolizumab is an IgG4-kappa humanized monoclonal antibody directed towards programmed cell death protein 1 (PD-1). PD-1, also known as CD 274 or B7-H1, is a costimulation receptor expressed by activated T cells. Binding of pembrolizumab to the PD-1 receptor prevents two immune-suppressing ligands, PD-L1 and PD-L2, from interacting with PD-1. Blocking of the PD-1 receptor by pembrolizumab thereby leads to inhibition of effector T cell proliferation, reduces cytotoxic activity, and induces apoptosis in tumor-infiltrating T cells and regulatory T cell expression [1].

This immunotherapy may now be used as a first-line agent for patients whose malignant cells have a PD-L1 expression or tumor proportion score (TPS) ≥ 1% and who do not harbor EGFR (epidermal growth factor receptor) or ALK (anaplastic lymphoma kinase) mutations [2]. Overall survival with the use of pembrolizumab correlates with increasing levels of PD-L1 expression [2].

Despite its survival benefits, it is also known for its immune-related adverse events, which affect various organ systems. Immune-related cardiotoxicity is a rare but often fatal complication. Cardiotoxicities associated with pembrolizumab include myocarditis, heart failure, sick sinus syndrome, cardiomyopathy, cardiac fibrosis, and cardiac arrest [3–7]. The following case describes a patient who developed complete heart block which appears to be temporally related to the use of the anti-PD-1 antibody, pembrolizumab.

## 2. Case Presentation

We provided care for a 67-year-old female with a past medical history of stage IV NSCLC metastatic to the adrenal gland, lymph nodes, and brain, complicated by a prior seizure for which she was on levetiracetam, hypertension on amlodipine, hyperlipidemia on simvastatin, hypothyroidism on levothyroxine, and depression on trazodone who presented to our Emergency Department as a transfer from the Cancer Center for bradycardia which was noted on routine vital sign assessment as she was about to get her second immunotherapy dose.

Her recent PET scan showed progression of her cancer in the mediastinum and supraclavicular area. Lymph node biopsy revealed a PD-L1 expression of 90%, and hence, she received her first infusion of pembrolizumab 200 mg intravenously three weeks prior to our encounter, as it is usually administered.

**Figure 1:**
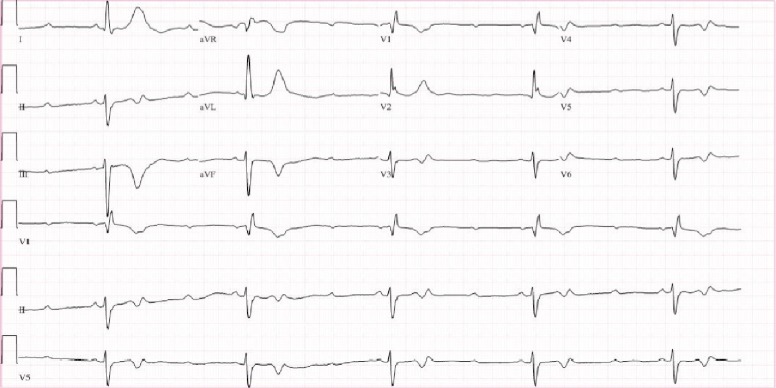
Progression of electrocardiogram tracings from the time of admission to immediately prior to transvenous pacer wire placement. Our patient was noted to have a heart rate of 31 bpm on admission, with initial EKG showing 2^nd^-degree AV block.

**Figure 2:**
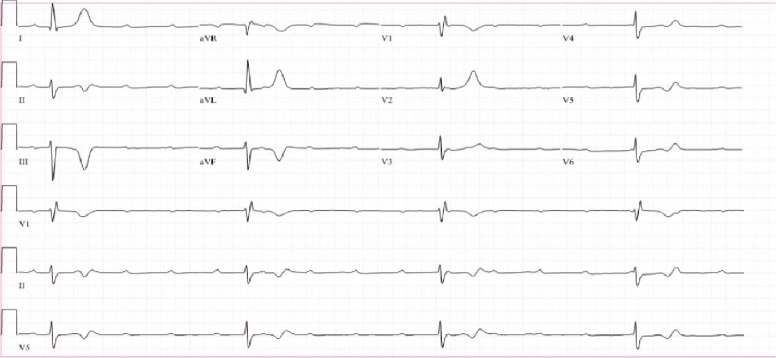
Progression of electrocardiogram tracings from the time of admission to immediately prior to transvenous pacer wire placement. Approximately three hours later, repeat EKG revealed complete heart block with ventricular rate of 22 bpm.

**Figure 3:**
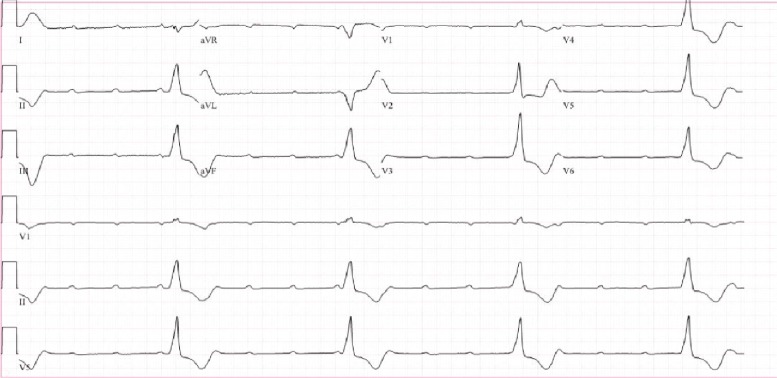
Progression of electrocardiogram tracings from the time of admission to immediately prior to transvenous pacer wire placement. EKG was repeated a few minutes later, showing complete heart block and idioventricular rhythm with a heart rate of 25 bmp immediately after starting a dobutamine infusion, at which point a transvenous pacer wire was emergently placed.

In the Emergency Department, she was initially asymptomatic with a heart rate of 30 beats per minute (bpm) as noted on telemetry monitoring and blood pressure of 121/63 mmHg. On admission, her EKG depicted Mobitz type 2 second-degree atrioventricular block (Figure 1). The electrophysiologist was immediately consulted with plans to place a permanent pacemaker the following morning. However, approximately three hours later, as she shifted in bed in order to place the bedpan beneath her, she began to feel lightheaded and her blood pressure dropped to 64/42 mmHg. Repeat EKG at this time showed complete heart block with a ventricular rate of 22 bpm (Figure 2). At this point, she was given a 500 cc bolus and dobutamine drip was initiated. EKG at this time showed complete heart block and idioventricular rhythm with a heart rate of 25 bpm (Figure 3), following which a temporary transvenous pacemaker was emergently placed overnight.

**Figure 4:**
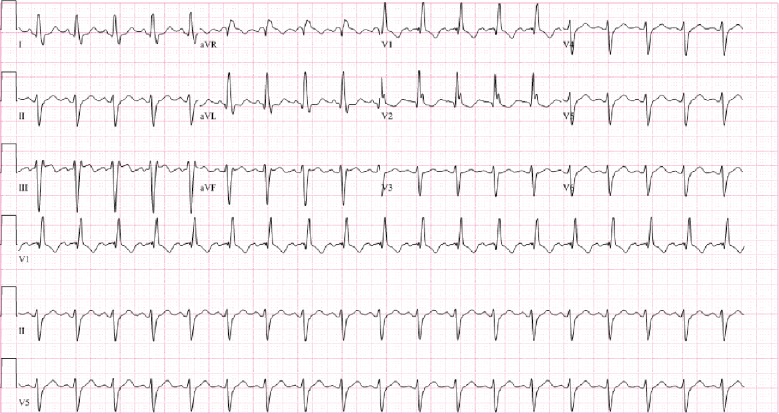
Baseline EKG which was obtained approximately a year and a half prior to presentation showed a left anterior fascicular block and a right bundle branch block.

Her labs including a basic metabolic panel and complete metabolic panel were unremarkable. Her magnesium level was 2.2 mg/dL and phosphorus level was 3.2 mg/dL. Troponin T was trended and was negative throughout her admission. Thyroid stimulating hormone level on admission was within normal range at 1.270 mU/mL. Her echocardiogram showed a LVEF of 60-65%. Her baseline EKG obtained approximately a year and a half prior to presentation showed a left anterior fascicular block and a right bundle branch block (Figure 4).

She underwent permanent pacemaker placement the following morning without complication. Prior to discharge, a chest X-ray, electrocardiogram, and device interrogation were performed. She was discharged home in a hemodynamically stable condition.

## 3. Discussion

Pembrolizumab is commonly associated with immune-related adverse events and is infrequently associated with cardiotoxicities such as myocarditis and heart failure. Conduction disease can be isolated or associated with coexisting myocarditis [8]. However, there are only two case reports in the literature so far in which patients presented with complete heart block. Interestingly, similar to the article by Katsume et al., our patient was receiving pembrolizumab after initial prior treatment with pemetrexed and bevacizumab for NSCLC. Katsume et al.'s patient was also found to have a high PD-L1 expression of >95% and received pembrolizumab at 200 mg 16 days prior to admission [7]. In other words, both patients were highly susceptible to the effects of pembrolizumab with their tumor cells having a high degree of expression (90% or greater), and both patients developed heart block the second time they received the immunotherapy, approximately 3 weeks after the first infusion. Our patient's prior chemotherapy with bevacizumab, a VEGF-A inhibitor, is also a recognized risk factor for immune checkpoint inhibitor-related cardiotoxicity [8].

Moreover, in the second published case of pembrolizumab-induced complete heart block by Jang and Stream, their patient who was being treated for metastatic renal cell carcinoma also presented with worsening exertional dyspnea precisely 3 weeks after his first infusion [9]. His PD-L1 expression level was not documented. Of note, the median interval from the initial administration of other commonly used immune checkpoint inhibitors, ipilimumab and nivolumab, to the development of myocarditis is 17 days [10].

It is also worthwhile to note that the patients in the two other cases were concurrently diagnosed with other immune-related adverse events such as biopsy-proven hepatitis (as presented by Katsume et al.) and myasthenia gravis (as presented by Jang and Stream), whereas our patient was asymptomatic on presentation and her laboratory testing was negative for elevations in troponin, creatine kinase, or liver enzymes. Thus, physicians should be mindful of immune-related adverse events in patients, irrespective of their symptoms and findings on blood work. Since pembrolizumab is administered every three weeks for a variety of indications, increased awareness of possible cardiotoxicity around the time of the second infusion is advised.

There are various proposed mechanisms attempting to explain immune checkpoint inhibitor-mediated arrythmias. Some of these theories include ventricular myocarditis with inflammation and fibrosis or inflammation of the His-Purkinje conduction system, both leading to reentry arrythmias [8]. Atrial or ventricular arrythmias secondary to inflammation without myocarditis, as well as atrial and ventricular arrythmias from functional cardiotoxicity without inflammation, are other possibilities [8]. Interestingly, genetic deletion of PD-1 in mice leads to cardiomyopathy that is caused by autoantibiodies against cardiac troponin I, which may be contributing to the cardiotoxicity caused by immune checkpoint inhibitors [10–12].

Ultimately, we recommend a higher degree of caution among all physicians when dealing with patients on immunotherapy. According to the clinical practice guidelines provided in the American Society of Clinical Oncology Journal for the management of immune-related adverse events in patients treated with immune checkpoint inhibitor therapy, holding checkpoint inhibitor therapy is recommended for all grades of cardiac complications and the appropriateness of rechallenging remains unknown [13]. Discontinuation of the immunotherapy with or without steroid treatment (1 to 2 mg/kg of prednisone) should be considered depending on the grade of toxicity as a therapeutic option [13]. In patients who do not immediately respond to high-dose corticosteroids, methylprednisolone 1 g daily can be attempted [13]. In some cases, further adding mycophenolate, infliximab, or antithymocyte globulin can be considered [13]. Cardiology consultation should always be obtained and transfer to the coronary care unit should be performed in the case of conduction abnormalities or troponemia [13].

Resuming immune checkpoint inhibitor therapy should be considered following multidisciplinary evaluation between cardiology, oncology, and cardiooncology if possible. In the case of our patient, her pembrolizumab was eventually restarted and she has so far tolerated nine additional cycles without any further discernable cardiac toxicities.
